# Relationships among parent and youth healthful eating attitudes and youth dietary intake in a cross-sectional study of youth with type 1 diabetes

**DOI:** 10.1186/1479-5868-10-125

**Published:** 2013-11-06

**Authors:** Tonja R Nansel, Denise L Haynie, Leah M Lipsky, Jing Wang, Sanjeev N Mehta, Lori MB Laffel

**Affiliations:** 1 Eunice Kennedy Shriver National Institute of Child Health and Human Development, Health Behavior Branch, Division of Intramural Population Health Research, NIH, DHHS, 6100 Executive Blvd., Rm. 7B13, MSC 7510, Bethesda, MD 20892-7510, USA; 2 Joslin Diabetes Center, Pediatric, Adolescent and Young Adult Section, Genetics and Epidemiology Section, One Joslin Place, Boston, MA 02215, USA

**Keywords:** Dietary intake, Social cognitive theory, Type 1 diabetes, Children, Adolescents

## Abstract

**Background:**

Constructs based on Social Cognitive Theory have shown utility in understanding dietary behavior; however, little research has examined these relations in youth and parents concurrently. Unique demands of dietary management among families of youth with type 1 diabetes (T1D) suggest the importance of investigation in this population. The purpose of this study was to develop and evaluate youth and parent measures of self-efficacy, outcome expectations, and barriers for healthful eating, and parent modeling of healthful eating, in a sample of youth with type 1 diabetes and their parents.

**Methods:**

Youth (n=252) ages 8–18 years with diabetes duration ≥1 year and parents completed self-report measures of healthful eating attitudes including self-efficacy, perceived barriers, positive and negative outcome expectations; youth reported parent modeling of healthful eating. Youth dietary intake from 3-day diet records was used to calculate the Healthy Eating Index 2005 and the Nutrient Rich Foods 9.3 index, measures of overall diet quality. The relations among parent and youth healthful eating attitudes, parent modeling, and youth diet quality were examined using structural equation modeling.

**Results:**

Internal consistency and test-retest reliability of the measures were acceptable. The structural equation model demonstrated acceptable fit (CFI/TLI=0.94/0.94; RMSEA=0.03), and items loaded the hypothesized factors. Parent modeling $\left(\hat{\beta }=.27,p=.02\right)$ and attitudes toward healthful eating (latent variable comprised of self-efficacy, barriers, outcome expectations) $\left(\hat{\beta }=.16,p=.04\right)$ had direct effects on youth diet quality. Parent modeling had a direct effect on youth attitudes $\left(\hat{\beta }=.49,p<.001\right)$; parent attitudes had an indirect effect on youth attitudes through parent modeling $\left(\hat{\beta }=.12,p,<.001\right)$. Youth attitudes were not associated with youth diet quality. Overall, the model accounted for 20% of the variance in child diet quality.

**Conclusions:**

Parent diet-related behaviors demonstrated an impact on youth attitudes and diet quality, suggesting the importance of family-based clinical and public health efforts to improve diet.

## Background

It is well-established that diet quality among US youth falls short of recommendations, with inadequate intake of fruits, vegetables, and whole grains, and excessive intake of sugar, highly processed foods, and saturated fat [1-3]. Eating behaviors during childhood and adolescence track into adulthood [4,5] and impact long-term health outcomes [6,7], underscoring the importance of understanding the determinants of these behaviors.

Dietary recommendations for youth with type 1 diabetes are the same as those for the general population, and families of youth with type 1 diabetes are encouraged to consume an overall healthful diet to optimize growth, maintain normal weight, and reduce risk for cardiovascular disease [8,9]. However, diet is uniquely relevant for this population due to the importance of attention to carbohydrate estimation for prandial insulin dosing. Both the amount and type of food consumed may impact postprandial blood glucose levels, and accurate estimation of carbohydrate intake is necessary for insulin dosing to optimize glycemic control.

Despite attention to diet as part of the diabetes management regimen, diet quality of youth with type 1 diabetes is similarly poor to that of the general youth population [10-13], or possibly worse, as children with type 1 diabetes consume a greater proportion of saturated fat [14,15]. The dramatic increase in obesity prevalence in youth with type 1 diabetes [16,17] and the high prevalence of cardiovascular risk factors among these youth [18,19] suggests the need for evaluating factors contributing to diet quality in this population. Given the role of parents both in facilitating diabetes management and decision-making regarding food in the home and family eating patterns, attention to both child and parent determinants of dietary intake appears warranted. As diabetes management involves attention to dietary intake, the potential role of parents as a gatekeeper of youth diet may potentially be greater than among the general population of youth. Yet we are aware of only one study addressing parental determinants of dietary intake among youth with type 1 diabetes, which demonstrated associations of parent education level, family income, and parents’ nutrition education with dimensions of youth dietary intake [20].

Research suggests the utility of social cognitive theory for understanding dietary intake among both youth and adults. Social cognitive theory indicates that behavior is determined by individual beliefs as well as socio-environmental factors [21]. Key predictive beliefs are outcome expectations (expected positive and negative outcomes of behaviors) and self-efficacy (perceived ability to perform behaviors), which develop from both vicarious and actual day-to-day experiences involving food, including modeling of eating behaviors by significant others. Outcome expectations and self-efficacy of healthy eating among children and their parents, which are potentially modifiable through skill development, experience, and reinforcement, are important targets for dietary behavior interventions. Youth fruit and vegetable intake are associated with self-efficacy [22-26] outcome expectations [27], and barriers [22,28] for fruit and vegetable consumption, as well as parent modeling [25,26,28-30]. Likewise, youth self-efficacy for decreasing “junk food” consumption is associated with lower fast food and snack intake [31]. Research in adults has shown associations between healthful eating self-efficacy [32-35], outcome expectations [32-34], and barriers [35] with indicators of diet quality including intake of fruits and vegetables, fat, and fiber. Furthermore, intervention studies in adults have demonstrated an increase in self-efficacy associated with increased fruit and vegetable intake [36] and decreased in waist circumference [37], as well as decreased barriers associated with decreased fat intake [38].

While much research has examined social cognitive constructs of adults as they relate to their own eating behaviors, limited research has examined these constructs as they pertain to parents’ attitudes regarding providing healthful foods for their families, which has implications for child diet quality given that parents serve a primary role in determining foods available to children in the home. Findings indicate an association of parents’ outcome expectations for purchasing fruits and vegetables with home availability [39], as well as between specific parent positive outcome expectations regarding fruit and vegetable consumption with child intake [40]. Additionally, as eating often takes place in the family context, parents may play a key role in the development of their child’s attitudes regarding healthful eating [41-43]. To our knowledge, previous research has not addressed the relationship of parent attitudes and modeling with child attitudes toward healthful eating. Gaining an understanding of the role of parents’ attitudes regarding providing healthful foods for their families, as well as the associations between parent and child attitudes and behaviors regarding healthful eating, is important for informing clinical and public health efforts to improve diet quality.

Although previous research has assessed social cognitive constructs as they pertain to intake of specific food groups, primarily fruits and vegetables, there is a need for measures of social cognitive constructs that address healthy eating more broadly. Evidence suggests that multiple aspects of dietary intake, including macronutrient intake and glycemic index, may influence glycemic control [44-47] and cardiovascular risk [44,48] in youth with type 1 diabetes. Both adolescents and adults generally understand fundamental principles of healthful eating, including which foods are more and less healthful [49,50]. Additionally, findings from focus groups among youth with type 1 diabetes [51,52] indicate that while youth widely perceive fruit and vegetable intake as relevant to healthful eating, they also understand the concept of healthful eating to encompass dietary behaviors such as fiber and whole grain intake, as well as limiting intake of nutrient-poor foods (e.g., snack foods, sweets). Such findings suggest the potential utility of examining social cognitive constructs as they relate to overall diet quality.

This paper describes the development and evaluation of youth and parent measures of self-efficacy, outcome expectations, barriers, and parent modeling of overall healthful eating in a sample of youth with type 1 diabetes and their parents. We focus on these constructs as they pertain to youth attitudes regarding their own eating behaviors, and parents’ attitudes regarding providing healthful foods for their families. This manuscript describes the psychometric properties of the measures as well as the structural equation model evaluating the association of youth and parent measures with youth diet quality. We hypothesized effects of parent attitudes toward healthful eating and modeling on youth attitudes, and effects of each on youth diet quality.

## Methods

### Design, sample, and procedures

Data were obtained from parent–child dyads recruited from a pediatric diabetes center in Boston, Massachusetts. Youth eligibility criteria included age 8 to 18 years, diagnosis of type 1 diabetes ≥ 1 year, daily insulin dose ≥ 0.5 units per kilogram, ability to communicate in English, and absence of chronic illness (particularly any GI disease such as celiac disease as the focus of this research was related to dietary behaviors) or medication that interferes significantly with diabetes management or glucose metabolism.

Medical record data were screened to identify eligible patients who were recruited to participate during a clinic visit by trained research staff. Families were invited to participate in a study aimed at understanding how families approach the diet of children and teens with type 1 diabetes. Parents and children age 18 years provided informed consent; children younger than 18 years provided assent. Survey measures were completed at the time of the clinic visit; diet records were completed by families following the clinic visit. To assess test-retest reliability, questionnaires were mailed to participants two weeks later with instructions to complete and return by mail. The study was approved by the Institutional Review Board of the clinical site. Of 455 eligible youth and their parents invited to participate; 302 youth from 291 families enrolled in the study. In families with multiple siblings enrolled, data from the sibling with the longest diabetes duration were retained. Of the 291 families enrolled, 259 completed the second survey administration, and 252 completed diet records.

### Measures

#### Social cognitive theory constructs

Self-report measures of self-efficacy, outcome expectations, barriers, and parent modeling were developed by the investigators. Parent and youth measures were not designed to reflect parallel constructs, but rather assessed their respective roles regarding youth’s dietary intake. The survey items were developed by a multi-disciplinary team consisting of behavioral scientists, nutrition scientists, pediatric endocrinologists, and certified diabetes educators. Item content and structure was informed by previous literature assessing these constructs as they pertained specifically to fruit and vegetable consumption [27,30,53]. Additionally, focus groups conducted with the population and clinical experience with the population further informed item content and selection of terminology. For some constructs, this yielded a greater number of items than optimal for a brief measure; however, all items were administered to allow item reduction to be guided by the item properties.

*Barriers* assessed environmental or skill impediments to healthful eating (child) or to providing healthful foods for the family (parent). Youth barriers included 8 items such as “Healthy food choices are not available at school” and “There is a lot of junk food at home”. Parent barriers included 7 items such as “There are not enough healthy food choices where I shop” and “I have very little time to prepare healthy meals”. Response options were on a 5-point Likert scale ranging from “strongly disagree” to “strongly agree”.

*Outcome expectations* assessed perceived positive and negative consequences of healthful eating (child) or providing healthful foods for the family (parent). Youth outcome expectations included 23 items such as “If I eat healthy foods like vegetables, fruits, whole grains, and beans… it would help me stay alert” (positive) and “…I wouldn’t get to eat the foods I really like” (negative). Parent outcome expectations included 18 items such as “If I served my family healthy foods like vegetables, fruits, whole grains, and beans… I would feel better about myself as a parent” (positive) and “…my family would complain” (negative). Response options were on a 5-point Likert scale ranging from “strongly disagree” to “strongly agree”.

*Self-efficacy* measured perceived ability to engage in healthful eating behaviors (child) or provide healthful foods for the family (parent). Youth self-efficacy included 22 items such as “I am sure I can…choose healthy foods at restaurants” and “…eat unhealthy foods less often”. Parent self-efficacy included 22 items such as “I am sure I can…make healthy meals that my family will enjoy” and “…limit the amount of junk food at home”. Response options were on a 5-point Likert scale ranging from “strongly disagree” to “strongly agree”.

*Parent modeling* of healthful eating was assessed by 9 items querying youth perceptions regarding their parents’ eating habits. Items included “When I was with my parents, they ate…vegetables”, “…fast food”, etc. Response options were on a 4-point Likert scale ranging from “almost never” to “almost always”.

Items with adequate variance and internal consistency were retained. We examined item skewness, percent of responses in the non-socially-desirable range (e.g., endorsement of low self-efficacy), item-to-total correlation, and change in Cronbach’s alpha if the item was removed from the scale. Items that were the most highly skewed, not internally consistent with the other items in the measure, or noted to be confusing to participants were eliminated. This resulted in retention of 8 youth barriers items, 16 youth outcome expectations items, 8 youth self-efficacy items, 8 youth report of parent modeling items, 7 parent barriers items, 14 parent outcome expectations items, and 11 parent self-efficacy items. The majority of items eliminated had low variance in responses. The retained items were included in the subsequent factor analyses using structural equation modeling, described below.

#### Dietary intake

The child’s usual dietary intake was estimated using three-day food records. Children and parents were jointly given a sample diet record and provided with detailed instructions on how to measure and report food and beverage intake, including specific details such as brand names or restaurants, and the use of measuring utensils when possible. Families were instructed to keep records on three consecutive days in one week, including two weekdays and one weekend day. Research staff reviewed the completed records upon receipt from the family to ensure completeness, and solicited missing information (e.g., brand names) from the family as needed. Nutrition Data System for Research software (NDSR; Nutrition Coordinating Center, University of Minnesota, Minneapolis, MN) was used to analyze the records. Two summary indices of diet quality, the Healthy Eating Index 2005 (HEI2005) and the Nutrition Rich Food 9.3 (NRF9.3) were calculated. The HEI-2005 [54,55] measures conformance to USDA dietary guidelines and is designed for use in both children and adults [54]. Possible scores range from 0 to 100; a score of 100 would indicate that all dietary guidelines were met. Because requirements for several food groups within the calculation of the HEI-2005 may be met through consumption of less healthful choices (e.g. fried vegetables), we also selected a measure of dietary nutrient density. Dietary nutrient density was measured using the NRF9.3, which is based on 9 nutrients to encourage (protein, fiber, vitamin A, vitamin C, vitamin E, calcium, iron, magnesium, and potassium) and 3 nutrients to limit (saturated fat, added sugar, and sodium), and calculated for total nutrient intake from food (not including dietary supplements) relative to energy intake [56]. NRF9.3 values of individual foods range, for example, from −56 for cola to 695 for spinach. NRF9.3 cutoff values indicative of good diet quality have not been specified; the mean NRF9.3 from analysis of NHANES data among persons age four and older was 13.3±0.5 [56].

#### Biomedical and demographic data

Biomedical data including hemoglobin A1c (HbA1c; reference range 4-6%; Tosoh 2.2 device, Tosoh Corporation, Foster City, CA), insulin regimen, and frequency of blood glucose monitoring were extracted from the medical records. Demographic characteristics were assessed by parent self-report.

### Analyses

Analyses were conducted in two steps. The first step was to evaluate psychometric properties of the measures of social cognitive constructs. Descriptive analyses were used to examine item distributions. To account for skewed distributions, three-category indicators were created by collapsing the three least socially desirable responses [57]. Items were all scored such that a higher score indicated attitudes supportive of healthful eating (e.g., a high score on barriers indicates fewer barriers). Cronbach’s alpha was used to assess internal consistency and Pearson correlation analyses were conducted to assess test-retest reliability. A series of confirmatory factor analyses (CFA) were conducted to confirm the measurement models for both child and parent measures of healthful eating attitude, with the three-category items as ordinal categorical indicators. Child healthful eating attitude was considered as a second-order factor, with child self-efficacy, positive and negative outcome expectations, and barriers as indicators. Similarly, parent healthful eating attitude was a second-order factor, modeled by parent self-efficacy, parent positive and negative outcome expectations, and barriers. In the second step, a structural equation model (SEM) was conducted to examine associations among child and parent healthful eating attitudes, parent modeling and youth diet quality. Child diet quality was modeled as a latent variable indicated by HEI-2005 and NRF9.3. Child and parent healthful eating attitude were included as two second-order factors [58]. Youth perception of parent modeling was included separately as a latent variable. Three sets of pathways were modeled to test the hypothesized direct and indirect relations between parent and youth healthful eating attitudes and parent modeling on youth diet quality:

1) From parent and youth healthful eating attitudes, and parent modeling on youth diet quality;

2) From parent healthful eating attitudes to child attitudes and to parent modeling; and

3) From parent modeling to child healthful eating attitudes.

Thus, the SEM model included direct effects of parent and child attitudes and parent modeling on diet quality, as well as of parent attitudes on child attitudes and parent modeling, and parent modeling on child attitudes. The CFAs and SEM were conducted with MPlus Version 6.11 (Muthen and Muthen, Los Angeles CA). Model fit was evaluated with respect to three goodness-of-fit statistics: comparative fit index (CFI), Tucker-Lewis fit index (TLI), and root mean square error of approximation (RMSEA). We considered a model with CFI >.90, TLI >.90, and RMSEA < .08 as reasonable or good fit [59].

## Results

### Sample characteristics

The sample was predominantly white and college-educated, with a majority of youth using insulin pump regimen (Table 1). The mean hemoglobin A1c indicated relatively good glycemic control [60]. The mean HEI2005 indicated youth diets are less than optimal according to the 2005 USDA Dietary Guidelines.

**Table 1:** Sample characteristics (n=252)

	**Mean ± SD or N (%)**
**Demographics**	
Age (years)	13.2 ± 2.8
Sex	
Female	122 (48.4)
Male	130 (51.6)
Race/ethnicity	
White, not Hispanic	231 (91.7)
Hispanic	9 (3.6)
Black	6 (2.4)
Other	6 (2.4)
Highest parent education level	
High school or equivalent	22 (8.7)
Junior college, technical, or some college	43 (17.1)
College degree	112 (44.4)
Graduate education	75 (29.8)
Family income (annual $)	
<30,000	22 (9.0)
30,000-49,999	17 (7.0)
50,000-69,999	31 (12.7)
70,000-99,999	52 (21.2)
100,000-149,999	57 (23.3)
>150,000	66 (26.9)
**Diabetes and health-related characteristics**	
Duration of diabetes (years)	6.3 ± 3.4
Regimen	
Injection	79 (31.3)
Pump	173 (68.7)
Daily frequency of blood glucose monitoring	5.4 ± 2.2
Hemoglobin A1c (%)	8.5 ± 1.3
Healthy Eating Index 2005	53.4 ± 11.0
Nutrient-Rich Food 9.3 score	20.8 ± 10.3

### Psychometric properties of the measures of social cognitive constructs

Confirmatory factory analysis of the items indicated 3 problematic items – 1 child outcome expectation item and 1 parent modeling item had low loadings with their respective designated factors (.17 and .26, respectively), and 1 parent outcome expectation item loaded across two factors (self-efficacy, modification index=103.3 and negative outcome expectations, modification index=85.3). After eliminating these items, both child (RMSEA = .047, CFI = .937 and TLI = .933) and parent (RMSEA = .066, CFI = .946 and TLI = .941) measurement models demonstrated good fit. All individual items loaded the specified factors (Table 2), and child and parent attitudes each comprised a second-order factor, with self-efficacy, positive and negative outcome expectation, and barriers as indicators. Internal consistency (alpha=.65-.87 for youth-report measures and .82-.89 for parent-report measures) and test-retest reliability (r=.57-.72 for youth-report measures and .65-.77 for parent-report measures) were acceptable to good (Table 2). The final measure, named the Healthful Eating Attitudes Scale, is provided in Additional file 1.

**Table 2:** Properties of the youth and parent social cognitive measures

	**Number of items**	**Item factor loadings**	**Cronbach’s alpha**	**Test-retest reliability**
**Youth-report**				
Self-efficacy	8	.71 - .84	.87	.61
Positive outcome expectations	5	.34 - .82	.75	.57
Negative outcome expectations	6	.73 - .88	.84	.63
Barriers	8	.32 - .87	.78	.72
Parent modeling	7	.37 - .64	.65	.71
**Parent-report**				
Self-efficacy	11	.61 - .91	.89	.76
Positive outcome expectations	6	.98 - .85	.82	.66
Negative outcome expectations	7	.47 - .90	.84	.77
Barriers	7	.64 - .96	.84	.65

### Associations among healthful eating attitudes, parent modeling and youth diet quality

**Figure 1:**
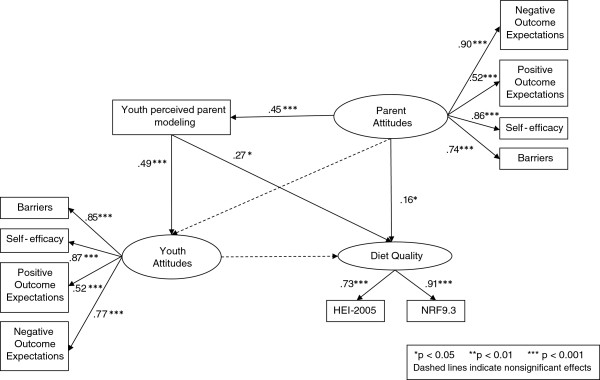
Structural equation model testing associations among child and parent healthful eating attitudes, parent modeling and youth diet quality.

The latent variables representing child and parent healthful eating attitudes and parent modeling were correlated with youth diet quality (Table 3). The full model (Figure 1) demonstrated good fit (CFI/TLI=0.94/0.94; RMSEA=0.03). Parent attitudes (higher self-efficacy and positive outcome expectations; lower negative outcome expectations and barriers) toward healthful eating had a direct positive effect on youth-perceived parent modeling (p<.001) and youth diet quality (p=.04), but were not directly associated with youth attitudes. In addition to the direct effect, parent attitudes had a significant indirect effect on diet quality (p=.03) through its effects on modeling (βˆ=12, p=02). Youth-perceived parent modeling of healthful eating had direct positive effects on youth attitudes (p<.001) and diet quality (p=.02). Youth attitudes toward healthful eating were not associated with diet quality. Overall, the model accounted for 20.0% of the variance in child diet quality.

**Table 3:** Correlations among latent variables of youth and parent social cognitive measures and diet quality

	**Diet quality**	** 1**	** 2**	** 3**	** 4**	** 5**	** 6**	** 7**	** 8**
1. Youth self-efficacy	.27***								
2. Youth negative outcome expectations	.16*	.64***							
3. Youth positive outcome expectations	.15*	.63***	.29***						
4. Youth barriers	.35***	.65***	.78***	.33***					
5. Youth-perceived parent modeling	.41***	.44***	.34***	.25**	.56***				
6. Parent self-efficacy	.29***	.29***	.22**	.12	.22***	.34***			
7. Parent negative outcome expectations	.26***	.25***	.23***	.05	.28***	.40***	.77***		
8. Parent positive outcome expectations	.16*	.21**	.16*	.12	.20**	.28***	.45***	.43***	
9. Parent barriers	.26***	.16**	.15*	.06	.20**	.39***	.63***	.70***	.36***

*p<.05

**p<.01

***p<.001

## Discussion

Findings from this study provide initial support for the utility of the newly-developed measures of parent and child healthful eating attitudes. Items loaded the hypothesized factors and the resulting scales demonstrated acceptable internal consistency and test-retest reliability. Youth and parent self-efficacy, outcome expectations, and barriers, and youth-perceived parent modeling were correlated with one another and with youth diet quality. These findings add to the body of research supporting the relevance of social cognitive theory for understanding determinants of youth dietary behaviors.

Few studies have examined determinants of dietary intake in youth with type 1 diabetes. Research in young children with type 1 diabetes has found mealtime interactions to be associated with adherence to meal carbohydrate content [61,62]; recent research in children and adolescents demonstrated associations of food preference and home availability with intake [63]. Contemporary insulin regimens allow for flexibility in dietary intake, and dietary intake of youth with type 1 diabetes is similar to that of the general population [13]. While nutrition counseling in type 1 diabetes care is necessarily focused on the youth’s dietary intake, particularly carbohydrate estimation, findings from this study highlight the importance of family-based interventions for promoting healthful eating among youth with type 1 diabetes.

This study extends previous research on social cognitive determinants of dietary intake by demonstrating the relationship of parent social cognitive attitudes and modeling of healthful dietary behavior to youth attitudes regarding healthful eating and dietary intake. Results presented suggest that parent attitudes and behaviors regarding healthful eating may impact youth diet not only through the shared food environment, but also by shaping the development of youth attitudes regarding healthful eating, highlighting the importance of parent eating behaviors in potentially shaping long-term youth dietary trajectories. Notably, parent attitudes toward and modeling of healthful eating were associated with youth diet quality in the structural equation model, while the effect of youth attitudes was not significant. These findings are consistent with research indicating that among youth, emotionally-based determinants may be stronger drivers of dietary behavior than cognitively-based ones [64].

Health communications targeting eating behavior often focus on its potential benefits, that is, positive outcome expectations. Our findings suggest that for healthful eating, negative outcome expectations – the anticipated negative consequences of the behavior – may be a stronger determinant of behavior than positive outcome expectations. It is perhaps not surprising that the anticipated negative consequences of healthful eating (e.g., deriving less enjoyment, being less convenient) may drive behavior more strongly than the anticipated positive consequences, as the former represent more concrete and proximal outcomes, and thus may be more salient in the context of day-to-day dietary decisions. As such, successfully achieving healthful dietary change may require greater attention to assessing and ameliorating perceived negative outcomes of healthful eating – that is, helping families find ways to make healthful eating satisfying, family-friendly, and time-efficient – as opposed to focusing primarily on the health benefits of the behavior.

The parent and youth social cognitive constructs assessed explained 20% of the variation in diet quality. While other factors such as environmental and cultural factors are known to be important determinants as well, self-efficacy, outcome expectations, barriers, and parent modeling may represent behavioral intervention targets with potential for meaningful impact. Only a few intervention studies in adults have examined whether change in self-efficacy or barriers mediates change in outcomes [36-38]; findings reported herein support the relevance of these constructs. Further investigation in the context of intervention studies is needed to more fully understand the extent to which change in these constructs mediate change in dietary behavior.

This study provides novel findings regarding the associations of both parent and youth social cognitive constructs regarding healthful eating to youth dietary intake. Strengths include a relatively large sample of youth with type 1 diabetes and the use of three-day diet records to assess dietary intake. The attention given to diet as an aspect of diabetes management may facilitate the reporting of dietary intake in families of youth with type 1 diabetes. Findings should be interpreted in light of study limitations, however. The sample was drawn from a single clinic with a limited number of minority and low-income families and a relatively large number of youth using insulin pump, with a mean HbA1c indicating relatively good glycemic control. Examination of the utility of these measures in broader samples is needed. Families choosing to participate may differ from the clinic population in dietary practices; however, dietary intake in this sample is consistent with previous research in type 1 diabetes [13] and US youth in general [2]. Data are cross-sectional, precluding determination of causality. Longitudinal research is required to examine more fully the relationships among parent and youth attitudes and youth dietary intake. While diet records are among the most reliable and valid measures of dietary intake, the task of completing food records may influence intake such that the records may not reflect usual intake. As with all self-report measures of intake, food records may be biased by social desirability [65]. However, the mean HEI-2005 in this sample was similar to that observed in a representative sample of US youth [2]. Parents and children were trained together in the completion of the diet records to address the developmental and practical realities of this population.

## Conclusions

Findings from this study suggest the utility of newly-developed measures of youth and parent social cognitive constructs regarding healthful eating and the provision of healthful meals for families of youth with type 1 diabetes. The study also uniquely demonstrates the influences of parent attitudes toward and modeling of healthful eating on youth attitudes and diet quality in this population at risk for adverse health outcomes. The poor dietary intake youth with type 1 diabetes, like that of US youth in general, has critical public health implications, and continued work in the development of effective behavioral nutrition interventions is warranted. Findings regarding the role of parents in impacting youth dietary intake suggest the importance of family-focused interventions to improve the diet quality of youth with type 1 diabetes.

## Competing interests

The authors declare that they have no financial or nonfinancial competing interests.

## Authors’ contributions

TRN led the development of the measures, conceived the research question, analyzed data, and wrote the manuscript. DLH provided substantive contribution to the development of the measures and reviewed/edited the manuscript. LML analyzed data and reviewed/edited the manuscript. JW provided statistical expertise, analyzed data, and reviewed/edited the manuscript. SNM contributed to development of the measures, collected data and reviewed/edited the manuscript. LMBL contributed to development of the measures, collected data and reviewed/edited the manuscript. All authors read and approved the final manuscript.

## Supplementary Material

Additional file 1 Healthful Eating Attitudes Scale. Click here for file
