# Supplementary material for: Relationships among parent and youth healthful eating attitudes and youth dietary intake in a cross-sectional study of youth with type 1 diabetes
Source: Int J Behav Nutr Phys Act. 2013 Nov 6;10:125. doi: 10.1186/1479-5868-10-125 (PMC3827889; doi:10.1186/1479-5868-10-125)
Supplement: Additional file 1 — Healthful Eating Attitudes Scale. [file 1479-5868-10-125-S1.docx]

**HEALTHFUL EATING ATTITUDES SCALE**

**YOUTH VERSION**

**PARENT MODELING**

**Think about the foods that you have seen your PARENTS / GUARDIANS eat during the PAST MONTH. Then,**

**for each of the foods below, choose the ONE answer that best describes how much of the time your PARENTS/ GUARDIANS ate that food when you were with them.**

|  | **When I was with my PARENTS / GUARDIANS, they ate…** | **Almost Never** | **Some-times** | **Often** | **Almost Always** |
| --- | --- | --- | --- | --- | --- |
|  | 1. Vegetables | ➀ | ➁ | ➂ | ➃ |
|  | 2. Fruit | ➀ | ➁ | ➂ | ➃ |
|  | 3. Salads | ➀ | ➁ | ➂ | ➃ |
|  | 4. Sweets (like candy, cake, or cookies) | ➀ | ➁ | ➂ | ➃ |
|  | 5. Fast food (like McDonald’s, Taco Bell, or KFC) | ➀ | ➁ | ➂ | ➃ |
|  | 6. Whole grains (like whole grain bread or brown rice) | ➀ | ➁ | ➂ | ➃ |
|  | 7. Salty snacks (like chips, pretzels, or crackers) | ➀ | ➁ | ➂ | ➃ |

**BARRIERS**

**The following statements are about healthy foods, like vegetables, fruits, whole grains, and beans. For each item, choose the ONE answer that best describes how much you agree or disagree with that statement.**

|  |  | **Strongly Disagree** | **Disagree** | **Neither Agree nor Disagree** | **Agree** | **Strongly Agree** |
| --- | --- | --- | --- | --- | --- | --- |
|  | 1. Healthy food choices are not available at school. | ➀ | ➁ | ➂ | ➃ | ➄ |
|  | 2. Choosing healthy foods is confusing. | ➀ | ➁ | ➂ | ➃ | ➄ |
|  | 3. I’m so busy that it’s hard to eat healthy. | ➀ | ➁ | ➂ | ➃ | ➄ |
|  | 4. Healthy food choices are not available at home. | ➀ | ➁ | ➂ | ➃ | ➄ |
|  | 5. I don’t know how to prepare healthy foods. | ➀ | ➁ | ➂ | ➃ | ➄ |
|  | 6. Healthy food choices don’t look good. | ➀ | ➁ | ➂ | ➃ | ➄ |
|  | 7. Healthy food choices don’t taste good. | ➀ | ➁ | ➂ | ➃ | ➄ |
|  | 8. There is a lot of junk food at home. | ➀ | ➁ | ➂ | ➃ | ➄ |

**OUTCOME EXPECTATIONS**

**Below are several statements about eating healthy foods, like vegetables, fruits, whole grains, and beans. For each statement, choose the ONE answer that best describes how much you agree or disagree with that sentence.**

|  | **If I eat healthy foods like vegetables, fruits,**  **whole grains, and beans…** | **Strongly Disagree** | **Disagree** | **Neither Agree nor Disagree** | **Agree** | **Strongly Agree** |
| --- | --- | --- | --- | --- | --- | --- |
|  | 1. I wouldn’t get to eat the foods I really like. | ➀ | ➁ | ➂ | ➃ | ➄ |
|  | 2. The food wouldn’t taste good. | ➀ | ➁ | ➂ | ➃ | ➄ |
|  | 3. I would have better blood sugar control. | ➀ | ➁ | ➂ | ➃ | ➄ |
|  | 4. It would help me be at a healthy weight. | ➀ | ➁ | ➂ | ➃ | ➄ |
|  | 5. I would have more energy. | ➀ | ➁ | ➂ | ➃ | ➄ |
|  | 6. It would be hard to find things to eat when out with my friends. | ➀ | ➁ | ➂ | ➃ | ➄ |
|  | 7. I would feel better. | ➀ | ➁ | ➂ | ➃ | ➄ |
|  | 8. Sharing food with my friends would be harder. | ➀ | ➁ | ➂ | ➃ | ➄ |
|  | 9. My friends would worry less about me. | ➀ | ➁ | ➂ | ➃ | ➄ |
|  | 10. There would be too many foods I couldn’t eat. | ➀ | ➁ | ➂ | ➃ | ➄ |
|  | 11. I would feel full longer after I eat. | ➀ | ➁ | ➂ | ➃ | ➄ |
|  | 12. I would be too different from my friends. | ➀ | ➁ | ➂ | ➃ | ➄ |
|  | 13. It would help me stay alert. | ➀ | ➁ | ➂ | ➃ | ➄ |
|  | 14. It would help me do better in sports or activities. | ➀ | ➁ | ➂ | ➃ | ➄ |
|  | 15. My parents would worry less about my diabetes. | ➀ | ➁ | ➂ | ➃ | ➄ |

**SELF-EFFICACY**

**The following statements are about healthy foods, like vegetables, fruits, whole grains, and beans. For each item, choose the ONE answer that best describes how confident you are that you can do that item.**

|  | **I am sure I can…** | **Strongly Disagree** | **Disagree** | **Neither Agree nor Disagree** | **Agree** | **Strongly Agree** |  |
| --- | --- | --- | --- | --- | --- | --- | --- |
|  | 1. Choose healthy foods at school | ➀ | ➁ | ➂ | ➃ | ➄ | |
|  | 2. Eat unhealthy foods less often | ➀ | ➁ | ➂ | ➃ | ➄ | |
|  | 3. Have my parent/guardian serve healthy foods to everyone in the family | ➀ | ➁ | ➂ | ➃ | ➄ | |
|  | 4. Prepare a healthy meal for myself | ➀ | ➁ | ➂ | ➃ | ➄ | |
|  | 5. Have others in the family not bring unhealthy snacks home | ➀ | ➁ | ➂ | ➃ | ➄ | |
|  | 6. Choose healthy foods when I eat at restaurants | ➀ | ➁ | ➂ | ➃ | ➄ | |
|  | 7. Choose healthy drinks (like milk or water) instead of soda or fruit drinks | ➀ | ➁ | ➂ | ➃ | ➄ | |
|  | 8. Eat healthy no matter what my friends are eating | ➀ | ➁ | ➂ | ➃ | ➄ | |

**HEALTHFUL EATING ATTITUDES SCALE**

**PARENT VERSION**

**BARRIERS**

**The following statements are about healthy foods, like vegetables, fruits, whole grains, and beans. For each item, choose the ONE answer that best describes how much you agree or disagree with that statement.**

|  |  | **Strongly Disagree** | **Disagree** | **Neither Agree nor Disagree** | **Agree** | **Strongly Agree** |
| --- | --- | --- | --- | --- | --- | --- |
|  | 1. There are not enough healthy food choices where I shop. | ➀ | ➁ | ➂ | ➃ | ➄ |
|  | 2. Choosing healthy foods is confusing. | ➀ | ➁ | ➂ | ➃ | ➄ |
|  | 3. I don’t know how to prepare healthy meals. | ➀ | ➁ | ➂ | ➃ | ➄ |
|  | 4. I have very little time to prepare healthy meals. | ➀ | ➁ | ➂ | ➃ | ➄ |
|  | 5. Healthy foods do not fit into my family’s food budget. | ➀ | ➁ | ➂ | ➃ | ➄ |
|  | 6. My family is so busy that it’s hard to eat healthy. | ➀ | ➁ | ➂ | ➃ | ➄ |
|  | 7. My spouse or other adults in my home will not eat healthy foods. □ *Check the box if there are no other adults at home.* | ➀ | ➁ | ➂ | ➃ | ➄ |

**OUTCOME EXPECTATIONS**

**Below are several statements about eating healthy foods, like vegetables, fruits, whole grains, and beans. For each statement, choose the ONE answer that best describes how much you agree or disagree with that sentence.**

|  | **If I served my family healthy foods like vegetables,**  **fruits, whole grains, and beans…** | **Strongly Disagree** | **Disagree** | **Neither Agree nor Disagree** | **Agree** | **Strongly Agree** |
| --- | --- | --- | --- | --- | --- | --- |
|  | 1. My family would not enjoy the food. | ➀ | ➁ | ➂ | ➃ | ➄ |
|  | 2. It would take too much time and effort. | ➀ | ➁ | ➂ | ➃ | ➄ |
|  | 3. It would help control my child’s blood sugar. | ➀ | ➁ | ➂ | ➃ | ➄ |
|  | 4. There would be too many foods we couldn’t eat. | ➀ | ➁ | ➂ | ➃ | ➄ |
|  | 5. It would help my family manage their weight. | ➀ | ➁ | ➂ | ➃ | ➄ |
|  | 6. My family would complain. | ➀ | ➁ | ➂ | ➃ | ➄ |
|  | 7. It would cost too much. | ➀ | ➁ | ➂ | ➃ | ➄ |
|  | 8. It would be too hard to eat out. | ➀ | ➁ | ➂ | ➃ | ➄ |
|  | 9. I would feel better about myself as a parent. | ➀ | ➁ | ➂ | ➃ | ➄ |
|  | 10. My family would not eat the food. | ➀ | ➁ | ➂ | ➃ | ➄ |
|  | 11. I would worry less about what my family eats. | ➀ | ➁ | ➂ | ➃ | ➄ |
|  | 12. My children would be more likely to make healthy food choices as adults. | ➀ | ➁ | ➂ | ➃ | ➄ |
|  | 13. I would worry less about my child with diabetes | ➀ | ➁ | ➂ | ➃ | ➄ |

**SELF-EFFICACY**

**The following statements are about healthy foods, like vegetables, fruits, whole grains, and beans. For each item, choose the ONE answer that best describes how confident you are that you can do that item.**

|  | **I am sure I can…** | **Strongly Disagree** | **Disagree** | **Neither Agree nor Disagree** | **Agree** | **Strongly Agree** |
| --- | --- | --- | --- | --- | --- | --- |
|  | 1. Make healthy meals that my family will enjoy | ➀ | ➁ | ➂ | ➃ | ➄ |
|  | 2. Pack a healthy lunch for my child | ➀ | ➁ | ➂ | ➃ | ➄ |
|  | 3. Find time to prepare healthy meals | ➀ | ➁ | ➂ | ➃ | ➄ |
|  | 4. Serve whole grain foods that my family will like | ➀ | ➁ | ➂ | ➃ | ➄ |
|  | 5. Make healthy choices when we eat out | ➀ | ➁ | ➂ | ➃ | ➄ |
|  | 6. Make healthy, good-tasting desserts from fruit | ➀ | ➁ | ➂ | ➃ | ➄ |
|  | 7. Plan meals ahead of time to make sure they include foods like vegetables, fruits, beans, and whole grains | ➀ | ➁ | ➂ | ➃ | ➄ |
|  | 8. Eat as healthy as I want my family to eat | ➀ | ➁ | ➂ | ➃ | ➄ |
|  | 9. Make healthier versions of our favorite foods | ➀ | ➁ | ➂ | ➃ | ➄ |
|  | 10. Limit the amount of junk food at home | ➀ | ➁ | ➂ | ➃ | ➄ |
|  | 11. Serve beans in a way my family will like | ➀ | ➁ | ➂ | ➃ | ➄ |
